# Effect of Dexmedetomidine-Assisted Intravenous Anesthesia on Gastrointestinal Motility in Colon Cancer Patients After Open Colectomy

**DOI:** 10.3389/fsurg.2022.842776

**Published:** 2022-02-25

**Authors:** Chaopeng Ou, Shiyang Kang, Ruifeng Xue, Jielan Lai, Yingjun Zhang

**Affiliations:** Department of Anesthesiology, Sun Yat-sen University Cancer Center, Guangzhou, China

**Keywords:** dexmedetomidine, intravenous anesthesia, open colectomy, gastrointestinal motility, gastrin

## Abstract

**Background:**

To explore the effect of dexmedetomidine (Dex)-assisted intravenous anesthesia on gastrointestinal motility in patients with colon cancer (CC) after open colectomy.

**Methods:**

A total of 102 patients with CC, undergoing open colectomy in our hospital from January 2018 to January 2020, were selected and randomly divided into an observation group (*n* = 51) and a control group (*n* = 51). The patients in the control group received a routine combination of intravenous and inhalation anesthesia (CIIA), while those in the observation group received a Dex-assisted CIIA. The systolic blood pressure (SBP), the diastolic blood pressure (DBP), heart rate (HR), and the mean arterial pressure (MAP) were compared at different time points between the two groups. In addition, the intraoperative general conditions, the dosage of anesthetics, and the recovery of gastrointestinal functions were also compared between the two groups. Moreover, before operation and at 24 h after operation, the levels of serum gastrin (GAS) and plasma motilin (MTL) were detected by radioimmunoassay, and the level of plasma cholecystokinin (CCK) was detected by an enzyme-linked immunosorbent assay. The incidence of gastrointestinal complications was recorded in both groups.

**Results:**

At T_1_-T_3_, the HR, SBP, DBP, and MAP levels were lower in both groups than those at T_0_. In addition, they were also lower in the observation group than those in the control group, showing significant differences (*p* < 0.05). The dosage of propofol and remifentanil in the observation group was lower than that in the control group, and there was a significant difference (*p* < 0.05). In the observation group, the postoperative first exhaust time, first defecation time, first ambulation time, and first feeding time were all earlier than those in the control group with significant differences (*p* < 0.05). After the operation, the observation group had higher levels of GAS and MTL but a lower level of CCK than the control group, and the differences were significant (*p* < 0.05). The incidence rate of gastrointestinal complications in the observation group (7.04%) was lower than that in the control group (19.61%), and there was a significant difference (χ^2^ = 4.346, *p* < 0.05).

**Conclusions:**

Dex-assisted intravenous anesthesia can facilitate the recovery of gastrointestinal motility, can regulate the levels of gastrointestinal hormones, and can stabilize the levels of hemodynamic indexes in patients with CC after open colectomy.

## Introduction

Colon cancer (CC) is a digestive tract malignancy that frequently occurs at the junction of the rectum and the sigmoid colon (1). It clinically manifests as abdominal pain, abdominal masses, changes in defecation habits, anemia, and gastrointestinal irritation and causes intestinal obstruction and intestinal perforation (2). Open colectomy, the current preferred treatment of CC, can radically clear and excise the tumor, producing a definite clinical efficacy (3). However, the gastrointestinal motility changes in some patients due to surgical trauma and intraoperative intravenous anesthesia, which inhibits the postoperative gastrointestinal motility, and in severe cases, affects the respiratory and circulatory function of the patient, thus negatively affecting the postoperative recovery (4). Dexmedetomidine (Dex) is a novel selective α2-adrenergic receptor agonist characterized by high intrinsic activity and short half-life (5). It can inhibit the release of catecholamines and sympathetic nervous excitability in the central sympathetic nervous system, without respiratory depression. Hence, it has been widely used for sedation during anesthesia or mechanical ventilation (6, 7). Previously, the effect of Dex in cancer surgery had been reported (8), but the effect of Dex in intravenous anesthesia on gastrointestinal motility in patients with CC after open colectomy has rarely been reported. In the present study, the effect of Dex-assisted intravenous anesthesia on gastrointestinal motility in patients with CC an after open colectomy was analyzed to provide a basis for the clinical treatment of patients with CC.

## Materials and Methods

### General Data

A total of 102 patients with CC undergoing open colectomy in our hospital from January 2018 to January 2020 were selected. The inclusion criteria were as follows: (1) patients meeting the diagnostic criteria for CC (9) and confirmed by the clinical-pathological examination; (2) those undergoing open colectomy; (3) those in American Society of Anesthesiologists (ASA) classes I-II and TNM stages I-II; (4) those who are not undergoing preoperative radiotherapy and chemotherapy; (5) those with an expected survival time >3 months; and 6) those who and whose families were fully informed of this study and had signed the informed consent. The exclusion criteria were as follows: (1) patients who are complicated with severe insufficiency in the heart, liver, or kidney, immune-related diseases, or systemic acute/chronic infectious diseases; (2) those with a history of allergy to drugs used in this study; or (3) those who are complicated with other malignancies and/or psychological or mental diseases. The patients were divided into an observation group (*n* = 51) and a control group (*n* = 51) using a random number table. General data, such as age, gender, and ASA class, had no significant differences between the two groups of patients (*p* > 0.05) (Table 1). This study was approved by the Hospital Ethics Committee.

**Table 1:** Comparison of general data between the two groups [*n* (%), χ ± s].

**Index**		**Observation group (*n* = 51)**	**Control group (*n* = 51)**	***χ* ^2^ */t***	***P***
Gender (n)	Male	34 (66.67)	30 (58.82)	0.671	0.413
	Female	17 (33.33)	21 (41.18)		
Age (Y)		55.02 ± 5.63	55.47 ± 5.71	0.401	0.689
ASA class (n)	I	29 (56.86)	27 (52.94)	0.158	0.691
	II	22 (43.14)	24 (47.06)		
TNM stage (n)	I	22 (43.14)	25 (49.02)	0.355	0.551
	II	29 (56.86)	26 (50.98)		
Tumor site (n)	Ileocecal junction	12 (23.53)	14 (27.45)	1.143	0.767
	Transverse colon	7 (13.73)	10 (19.61)		
	Ascending colon	9 (17.65)	7 (13.73)		
	Left hemicolon and sigmoid colon	23 (45.10)	20 (39.22)		
Maximum diameter of tumor (cm)		5.73 ± 1.54	5.66 ± 1.38	0.242	0.809

### Anesthesia Methods

The patients in the control group received a routinely combined intravenous and inhalation anesthesia (CIIA). Under routine electrocardiograph monitoring, intravenous access was established. Midazolam (Jiangsu Nhwa Pharmaceutical Co., Ltd., NMPN H20143222) at.03 mg/kg, fentanyl (Yichang HumanWell Pharmaceutical Co., Ltd., NMPN H42022076) at 3 μg/kg, propofol injection (Xi'an Libang Pharmaceutical Co., Ltd., NMPN H20040300) at 1.5 mg/kg, and cisatracurium besilate (Jiangsu Hengrui Pharmaceutical Co., Ltd., NMPN H20060869) at 0.6 mg/kg were used for the intravenous target-controlled infusion to induce anesthesia until muscular relaxation, followed by tracheal intubation. Sevoflurane (Hebei Yipin Pharmaceutical Co., Ltd., NMPN H20173156) was intravenously infused at 0.5–2% to maintain anesthesia, propofol injection was infused at 0.07 μg/kg/min with an additional 1.5 μg every 30 min, and cisatracurium besilate was additionally supplemented at 0.3 mg/kg every 30 min until 20 min before the end of the operation.

The patients in the observation group received a Dex-assisted CIIA. At 10 min before anesthesia induction, a loading dose of Dex (Jiangsu Hengrui Pharmaceutical Co., Ltd., NMPN H20090248) was pumped at 1 μg/kg, and the methods of anesthesia induction and maintenance were the same as those in the control group. After the start of the operation, Dex was continuously pumped at 1.5 μg/kg/h until 30 min before the end of the operation. Open colectomy was conducted under general anesthesia in both groups.

### Observation Indexes

#### Changes in SBP, DBP, HR, and Mean Arterial Pressure (MAP) at Different Time Points

The changes in SBP, DBP, HR, and MAP were recorded in both groups before anesthesia induction (T_0_), at the time of intubation (T_1_), at the time of extubation (T_2_), and 5 min after extubation (T_3_).

#### Intraoperative General Conditions and Recovery of Postoperative Gastrointestinal Function

The intraoperative blood loss, intraoperative infusion volume, operation time, and dosage of propofol and remifentanil were compared between the two groups. The postoperative first exhaust time, the first defecation time, the first ambulation time, and the first feeding time were recorded in the two groups.

#### Indexes of Gastrointestinal Function

Before the operation and at 24 h after operation, 5 ml of fasting venous blood was drawn from each patient in the two groups in the early morning. The serum and plasma were separated by centrifugation and stored at −30°C for later use. The levels of serum gastrin (GAS) and plasma motilin (MTL) were detected by radioimmunoassay using kits manufactured by Shanghai X-Y Biotechnology Co., Ltd., (Shanghai, China), and the level of plasma cholecystokinin (CCK) was detected by ELISA using kits manufactured by Shanghai Enzyme Research Biotechnology Co., Ltd. (Shanghai, China) in strict accordance with the instructions (10, 11).

### Gastrointestinal Complications

The incidence of gastrointestinal complications, such as abdominal pain, abdominal distension, nausea, and intestinal obstruction, was recorded in the two groups.

### Statistical Analysis

The Statistical Product and Service Solutions (SPSS) 22.0 software (IBM, Armonk, NY, USA) was used for data analysis. Measurement data were expressed as (χ ± s). Independent-sample (two-sample) *t*-test was used to compare the intergroup difference without time factors, and repeated measures of ANOVA was done to compare the intergroup difference with time factors. Enumeration data were expressed as rate, and χ^2^ test was performed for the difference between the two groups. Two-sided *p* < 0.05 was considered statistically significant.

## Results

### Comparison of Changes in HR, SBP, DBP, and MAP Between the Two Groups at Different Time Points

At T_0_, HR, SBP, DBP, and MAP levels had no statistically significant differences between the two groups (*p* > 0.05). At T_1_-T_3_, HR, SBP, DBP, and MAP levels were lower in both groups than those at T_0_, and they were also lower in the observation group than those in the control group (*p* < 0.05) (Table 2).

**Table 2:** Comparison of changes in heart rate (HR), systolic blood pressure (SBP), diastolic blood pressure (DBP), and mean arterial pressure (MAP) between the two groups at different time points (χ±s).

**Index**	**Time point**	**Observation group** **(*n* = 51)**	**Control group** **(*n* = 51)**
HR (beats/min)	T_0_	105.43 ± 5.27	106.14 ± 5.08
	T_1_	92.58 ± 6.25	98.62 ± 5.47
	T_2_	88.65 ± 6.89	96.28 ± 6.15
	T_3_	82.52 ± 6.43	90.49 ± 6.96
*F*_time_ */P*		85.59/ <0.001	
*F*_intergroup_ */P*		178.40/ <0.001	
*F*_interaction_ */P*		7.73/ <0.001	
SBP (mmHg)	T_0_	120.33 ± 10.21	120.81 ± 10.36
	T_1_	113.28 ± 9.15	119.07 ± 9.14
	T_2_	104.50 ± 8.74	112.43 ± 9.55
	T_3_	93.85 ± 8.36	103.89 ± 10.13
*F*_time_ */P*		41.68/ <0.001	
*F*_intergroup_ */P*		102.90/ <0.001	
*F*_interaction_ */P*		4.78/0.002	
DBP (mmHg)	T_0_	88.72 ± 8.44	89.15 ± 8.51
T_0_	T_1_	83.24 ± 9.18	87.64 ± 8.92
T_1_	T_2_	72.91 ± 9.75	83.27 ± 9.13
T_2_	T_3_	65.83 ± 10.26	72.58 ± 9.69
*F*_time_ */P*		35.83/ <0.001	
*F*_intergroup_ */P*		90.89/ <0.001	
*F*_interaction_ */P*		5.17/0.001	
MAP (mmHg)	T_0_	126.87 ± 12.43	130.45 ± 12.19
	T_1_	129.95 ± 13.08	122.76 ± 13.05
	T_2_	127.89 ± 12.52	121.15 ± 12.58
	T_3_	112.46 ± 10.19	104.95 ± 9.82
*F*_time_ */P*		14.03/ <0.001	
*F*_intergroup_ */P*		57.79/ <0.001	
*F*_interaction_ */P*		5.07/0.001	

### Comparison of Intraoperative General Conditions and Dosage of Anesthetics Between the Two Groups

The intraoperative blood loss, the intraoperative infusion volume, and othe peration time had no statistically significant differences between the two groups (*p* > 0.05). The dosage of propofol and remifentanil in the observation group was lower than that in the control group (*p* < 0.05) (Table 3).

**Table 3:** Comparison of intraoperative general conditions and dosage of anesthetics between the two groups (*n* = 51, $\bar{x}$ ± s).

**Group**	**Intraoperative blood loss (mL)**	**Intraoperative infusion volume (L)**	**Operation time (h)**	**Propofol (g)**	**Remifentanil (mg)**
Observation group	291.35 ± 17.29	1.33 ± 0.12	2.94 ± 0.35	0.83 ± 0.13	1.669 ± 0.36
Control group	292.73 ± 19.04	1.39 ± 0.27	3.08 ± 0.41	1.05 ± 0.15	2.51 ± 0.49
*t*	0.383	1.450	1.855	7.915	9.631
*P*	0.702	0.150	0.067	<0.001	<0.001

### Comparison of Recovery of Postoperative Gastrointestinal Function Between the Two Groups

In the observation group, the postoperative first exhaust time, the first defecation time, the first ambulation time, and the first feeding time were all earlier than those in the control group (*p* < 0.05) (Table 4).

**Table 4:** Comparison of recovery of postoperative gastrointestinal function between the two groups (*n* = 51, $\bar{x}$ ± s).

**Group**	**First exhaust time (h)**	**First defecation time (d)**	**First ambulation time (h)**	**First feeding time (d)**
Observation group	37.42 ± 4.65	2.14 ± 0.36	29.81 ± 3.55	3.02 ± 0.43
Control group	51.23 ± 5.34	3.58 ± 0.55	40.13 ± 5.12	3.59 ± 0.47
*t*	13.928	15.644	11.829	6.390
*P*	<0.001	<0.001	<0.001	<0.001

### Comparison of Indexes of Gastrointestinal Function Between the Two Groups Before and After the Operation

No statistically significant differences were found in the levels of GAS, MTL, and CCK between the two groups before operation (*p* > 0.05). After the operation, the levels of GAS and MTL rose, while the level of CCK declined in the two groups compared with those before the operation (*p* < 0.05). After the operation, the observation group had significantly higher levels of GAS and MTL but a significantly lower level of CCK than the control group (*p* < 0.05) (Table 5).

**Table 5:** Comparison of indexes of gastrointestinal function between the two groups before and after the operation (*n* = 51, $\bar{x}$ ± s).

**Group**	**GAS (pg/mL)**		**MTL (pg/mL)**		**CCK (pg/mL)**	
	**Before operation**	**After operation**	**Before operation**	**After operation**	**Before operation**	**After operation**
Observation group	42.54 ± 4.87	83.42 ± 6.56	253.19 ± 18.39	316.22 ± 26.94	58.56 ± 6.77	37.21 ± 4.34
Control group	43.19 ± 4.35	72.18 ± 6.14	251.58 ± 19.74	293.69 ± 24.87	57.94 ± 6.50	45.69 ± 5.28
*t*	0.711	8.934	0.426	4.388	0.472	8.860
*P*	0.479	<0.001	0.671	<0.001	0.638	<0.001

### Comparison of Incidence of Gastrointestinal Complications Between the Two Groups

In the observation group, there were 2 cases of abdominal distension, 1 case of abdominal pain, and 2 cases of nausea after the operation. In the control group, there were 4 cases of abdominal distension, 1 case of abdominal pain, 3 cases of nausea, and 2 cases of intestinal obstruction after the operation. It can be seen that the incidence rate of gastrointestinal complications in the observation group (7.04%) was lower than that in the control group (19.61%) (χ^2^ = 4.346, *p* < 0.05).

## Discussion

Colon cancer (CC) is a gastrointestinal tract malignancy derived from the colonic mucosal epithelium, manifested as varying degrees of abdominal distension, indigestion, and changes in defecation habits in most patients (12). Radical surgery is an effective treatment means for CC, but some patients suffer from intraoperative hemodynamic fluctuations and enhanced sympathetic nervous excitability due to intestinal obstruction and the intravenous anesthetics used, resulting in postoperative gastrointestinal dysfunction (4, 13). Dex is a fast-onset and short-acting α2-adrenergic receptor agonist with sedative and analgesic effects but no respiratory depression, which can effectively lower sympathetic nervous excitability and restore gastrointestinal function (14).

In this study, at T1-T3, the HR, SBP, DBP, and MAP levels were lower in both groups than those at T0, and they were also lower in the observation group than those in the control group, suggesting that Dex can effectively improve both blood pressure and HR of patients with CC undergoing open colectomy. During intravenous anesthesia, intubation and extubation can cause irritation of varying degrees to patients, leading to fluctuations in blood pressure, HR, and other hemodynamic indexes. Dex can, through binding to α2 receptors, inhibit the further outflow of sympathetic media, thereby weakening the sympathetic nervous excitability during intubation and extubation and keeping hemodynamic indexes stable in patients with CC undergoing open colectomy (15). In this study, the intraoperative blood loss, the intraoperative infusion volume, and the operation time had no significant differences between the two groups, and the dosage of propofol and remifentanil in the observation group was lower than that in the control group, indicating that the dosage of anesthetics can be reduced in Dex-assisted intravenous anesthesia in open colectomy, further ameliorating gastrointestinal dysfunction caused by anesthetics. Previous evidence showed that Dex can stabilize the hemodynamic indexes of patients undergoing hepatectomy. In addition, it can also effectively relieve the stress response in laparoscopic gastrectomy and reduce the dosage of propofol and remifentanil, which is consistent with the results in this study (16, 17). The above findings confirm that Dex-assisted intravenous anesthesia can better stabilize the hemodynamic indexes of patients with CC undergoing open colectomy and effectively reduce the dosage of anesthetics with high safety.

In this study, the postoperative first exhaust time, the first defecation time, the first ambulation time, and the first feeding time in the observation group were all earlier than those in the control group, suggesting that Dex-assisted intravenous anesthesia can promote the recovery of gastrointestinal function in patients with CC after open colectomy. Due to stress, surgical trauma, and anesthetics, patients with CC undergoing open colectomy are prone to intestinal motility disorders, resulting in postoperative gastrointestinal dysfunction. Dex is able to maintain hemodynamic stability, alleviate inflammatory and stress responses, reduce the dosage of intravenous anesthetics, promote intestinal microcirculation perfusion, and protect the intestinal barrier function, contributing to the recovery of postoperative gastrointestinal motility (18, 19). Previous evidence showed that Dex-combined anesthesia exerts a protective effect on the intestinal barrier function of patients with acute intestinal obstruction, which is consistent with the results in this study, indicating that Dex-assisted intravenous anesthesia can facilitate the recovery of gastrointestinal function in patients with CC after open colectomy. Besides, GAS, MTL, and CCK are all important gastrointestinal hormones. GAS can promote gastric emptying through stimulating gastric acid secretion, MTL can enhance gastrointestinal motility, and CCK can suppress gastric emptying by inhibiting the contraction of the esophageal sphincter (20). In this Study, the observation group had higher levels of GAS and MTL but had a lower level of CCK than the control group after the operation, demonstrating that Dex can regulate the levels of gastrointestinal hormones in patients with CC after open colectomy, thereby improving the gastrointestinal function. In addition, the incidence rate of gastrointestinal complications (abdominal distension, abdominal pain, nausea, and intestinal obstruction) in the observation group was lower than that in the control group, further confirming that Dex-assisted intravenous anesthesia can boost the recovery of gastrointestinal motility with high safety in patients with CC after open colectomy.

## Conclusions

In conclusion, Dex-assisted intravenous anesthesia can facilitate the recovery of gastrointestinal motility, stabilize the levels of hemodynamic indexes, and regulate the levels of gastrointestinal hormones in patients with CC after open colectomy, with high safety. However, there were deficiencies in this study. For example, the sample size was limited, and the effect of non-effect dose of Dex on gastrointestinal motility in patients with CC after open colectomy was not explored and analyzed. Therefore, the sample size remains to be expanded for validation in the future.

## Data Availability Statement

The original contributions presented in the study are included in the article/supplementary material, further inquiries can be directed to the corresponding author.

## Ethics Statement

The studies involving human participants were reviewed and approved by the Ethics Committee of Sun Yat-sen University Cancer Center. The patients/participants provided their written informed consent to participate in this study.

## Author Contributions

CO, SK, and YZ designed the study and prepared the manuscript. CO, SK, and RX collected the data. YZ and JL analyzed the data. All authors read and approved the final manuscript. All authors contributed to the article and approved the submitted version.

## Conflict of Interest

The authors declare that the research was conducted in the absence of any commercial or financial relationships that could be construed as a potential conflict of interest.

## Publisher's Note

All claims expressed in this article are solely those of the authors and do not necessarily represent those of their affiliated organizations, or those of the publisher, the editors and the reviewers. Any product that may be evaluated in this article, or claim that may be made by its manufacturer, is not guaranteed or endorsed by the publisher.
